# A patient with agranulocytosis following the discontinuation of methimazole treatment for 4 months: A case report

**DOI:** 10.3892/etm.2014.1817

**Published:** 2014-06-30

**Authors:** XIAO-SU BAI, JING-HAI LIU, SHAO-MEI XIAO

**Affiliations:** Department of Metabolism and Endocrinology, People’s Hospital Of New District Longhua Shenzhen, Shenzhen, Guangdong 518109, P.R.China

**Keywords:** agranulocytosis, methimazole, hyperthyroidism

## Abstract

Agranulocytosis is a rare and serious adverse effect of antithyroid drugs (ATD), in particular methimazole (MMI), and usually develops within 3 months following the start of uninterrupted ATD treatment. Agranulocytosis may also develop for the first time following interruption and subsequent resumption of the same ATD treatment. In this case report, a 27-year-old female, who was treated for thyrotoxicosis with MMI, developed agranulocytosis following the discontinuation of MMI treatment for four months. To the best of our knowledge, this is the first study to report this. The aim of this report is to increase the awareness of physicians of the onset of agranulocytosis when MMI is discontinued, and to demonstrate that MMI should be used with caution.

## Introduction

Agranulocytosis, a rare antithyroid drug-induced complication that may be life threatening, is characterized by a marked reduction in the number of circulating granulocytes and a neutrophil count of <0.5×10^9^/l (1). Agranulocytosis usually occurs within the first 2–3 months of treatment (2); however, certain cases have demonstrated that agranulocytosis may occur following long-term treatment (3). Previously reported cases of agranulocytosis have been due to continuous antithyroid drug (ATD) treatment; however, in the present study a case of ATD-induced agranulocytosis occurring following the discontinuation of methimazole (MMI) treatment for 4 months is presented. This report, to the best of our knowledge, is the first time that a case of agranulocytosis following discontinued MMI has been reported.

## Case report

This study was approved by the Ethics Committee of People’s Hospital Of New District Longhua Shenzhen (Shenzhen, China). Written informed consent was obtained from the patient. A 27-year-old female was admitted to People’s Hospital Of New District Longhua Shenzhen in May 2013 with complaints of fever, sore throat and hypodynamia for three days. Three days prior to admission, the patient started to feel cold and had a fever, although the temperature was not taken, and the patient experienced a sore throat, dizziness and hypodynamia with unknown causes. Two days prior to admission, the patient started vomiting twice a day; the amount of vomit was unknown. The patient did not have a headache, chest distress, cardiopalmus, abdominal pain, diarrhea, cough or expectoration. One day prior to admission, the patient visited the local Community Health Service, and the results from the laboratory tests were as follows: white blood cell (WBC) count, 1.28×10^9^/l; neutrophils, 0.04×10^9^/l; hemoglobin, 143 g/l; and platelets, 231×10^9^/l. The patient was prescribed cefathiamidine, vitamin C intravenous drip, once daily (qd) and 4 compound Coptidis Rhizome capsules, twice daily (bid). However, the patient’s condition did not improve and the patient was transferred to People’s Hospital Of New District Longhua Shenzhen the following day.

**Figure 1:**
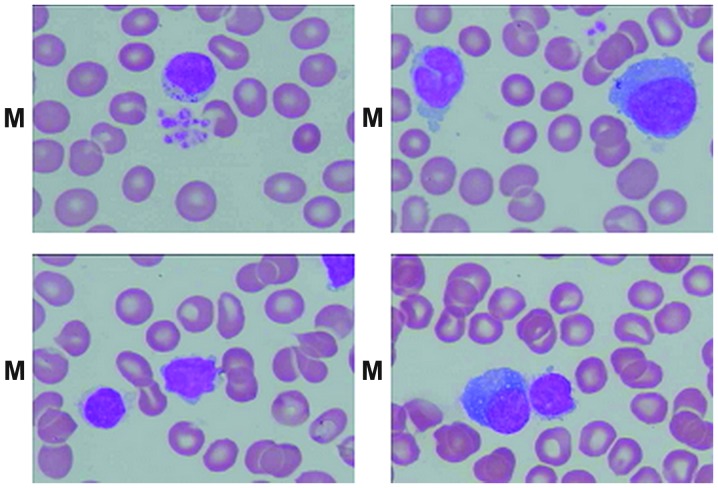
Bone marrow shows rare granulocytic precursors (magnification, ×1,000). M, bone marrow smears. Upper left figure: lymphocytes, platelets; Upper right: Neutrophilic stab granulocyte, atypical lymphocytes; Bottom left: Lymphocytes; Bottom right: Atypical lymphocytes, late erythroblast.

The patient had a history of hyperthyroidism. Four years previously, the patient had been diagnosed with hyperthyroidism, and prescribed methimazole [MMI; 10 mg; three times a day (tid)] and propanolol (10 mg; tid). After one month, the dose of MMI was gradual reduced to 10 mg, bid; after 4 months, MMI was reduced to 10 mg, qd; and after 6 months, MMI was reduced to 5 mg, qd, and this dosage was maintained for 18 months. The propanolol treatment was initiated to maintain the heart rate; when the heart rate returned to normal, the propanolol treatment was discontinued. During the initial treatment, blood samples were analyzed once a week; following this, they were analyzed once a month, and the results of these blood tests were normal. The course of treatment was 2 years. The patient made a full recovery from hyperthyroidism and MMI was discontinued. Six months previously, the patient’s hyperthyroid symptoms returned, and the patient was prescribed 10 mg MMI, tid and 10 mg propanolol, tid, at a local hospital. After two months of treatment, the patient refused to continue taking the medicine as she considered it to be ineffective. The patient had no history of hypertension, diabetes, kidney disease or other chronic diseases, or tuberculosis, hepatitis, typhoid fever or other infectious diseases. In addition, the patient had no history of trauma surgery and no a history of medicine or food allergies. The previous vaccination history of the patient was unknown. On admission to hospital, the results from the physical examination were as follows: temperature, 38.6°C; pulse, 138 beats/min; respiratory rate, 30 breaths/min and blood pressure, 137/94 mmHg. No proptosis was observed and general superficial lymph node enlargement was not palpable. The patient had pharyngeal congestion, a 2-fold enlarged thyroid, bilateral tonsil enlargement of II degree, and visible purulent secretions on the left side of the tonsil. The bilateral thyroid enlargement was of II degree, but no vascular murmurs were audible. The patient had a mild hand tremor; however, no rash or jaundice was observed. The results from the blood tests showed an absolute neutrophil count of zero and a total WBC count of 0.50×10^9^/l. The differential count showed 2.2% neutrophils (reference range, 45.0–73.0%), 95% lymphocytes (reference range, 20.0–40.0%), 0.7% monocytes (reference range, 5.0–11.0%), 0% eosinophils (reference range, 0.5–5.0%) and 0.8% basophils (reference range, 0.0–1.0%). The hemoglobin concentration and platelet count were normal at 134 g/l (reference range, 110–150 g/l) and 190×10^9^/l (reference range, 100–360×10^9^/l), respectively. The erythrocyte sedimentation rate was 33 mm/h (reference range, 0–10 mm/h). Results from the marrow biopsy showed hyperplasia with karyocyte proliferation. The granulocyte precursors were normal; however, the ratio of granulocyte to erythrocyte (G:E) counts was low. The ratio of leukomonocytes was 79% (reference range, 15.74–29.82%) and the heterology ratio of leukomonocytes was 3% (Fig. 1). Thyroidal function tests were as follows: free T3, 12.58 pmol/l (reference range, 3.80–6.00 pmol/l); free T4>80.45 pmol/l (reference range, 7.86–14.41 pmol/l); and thyroid-stimulating hormone (TSH), 0.01 mIU/l (reference range, 0.34–5.60 mIU/l). The results from the ultrasonograph showed diffuse goiter and normal liver. The results from the electrocardiogram (ECG) revealed sinus tachycardia, and chest X-ray showed no abnormalities. Based on these results, the patient was diagnosed with acute agranulocytosis, diffuse toxic goiter, thyroid crisis and acute tonsillitis.

On admission to People’s Hospital Of New District Longhua Shenzhen, the patient was prescribed oxygen, ECG, hydrocortisone to improve the patient’s stress response, propranolol to inhibit T4 transformation into T3 and to inhibit excitatory effects on the heart, and intravenous antibiotics (piperacillin and tazobactam) to control infection. Granulocyte-colony stimulating factor (GCSF) was administered to raise neutrophil numbers, with an initial dose of 100 μg/day. Due to poor response and sustained agranulocytosis, the dosage was increased to 300 μg/day after six days. After two days, the neutrophil count was increased to 0.02×10^9^/l; after four days, the neutrophil count was increased to 0.07×10^9^/l; and after six days, the neutrophil count was increased to 2.46×10^9^/l (Table I). After 10 days of treatment, the neutrophil count was increased to 4.53×10^9^/l, and the patient’s symptoms were generally improved; the patient no longer had a fever, sore throat or hypodynamia. When discharged from the hospital, the patient was prescribed oral I-131.

## Discussion

ATDs, in particular thioamides, including MMI, propylthiouracil and carbimazole, have adverse hematological effects, ranging from mild leucopenia to agranulocytosis and aplastic anemia. The incidence of ATD-induced agranulocytosis in patients with hyperthyroidism is rare; however, serious and potentially life-threatening adverse effects may occur, mainly due to severe systemic infection, if appropriate medical intervention is not administered immediately. ATD-induced agranulocytosis usually occurs within 2 or 3 months of ATD treatment; however, in certain cases this may be delayed. By reviewing previous studies, it was identified that the previously reported cases all occurred following continuous ATD treatment. In the present study, a case of ATD-induced agranulocytosis following treatment with MMI that was discontinued for four months is presented.

ATD-induced agranulocytosis is mediated by a variety of mechanisms, including direct toxic effects and immunological reactions. ATDs readily penetrate the marrow, affecting oxygen and glucose utilization of leukocytes through their oxidized metabolites (4). Toxic effects require between 20 and 40 days of exposure, and the onset is insidious. It is usually dose- and concentration-dependent (5), and is associated with continuous administration. However, the present report is not in accordance with this. In addition, damage to stem cells or granulocytic precursors in the bone marrow prevents the differentiation of granulocytes, without affecting the peripheral pool of neutrophils.

Wall *et al* (6) observed that *in vitro* peripheral lymphocyte transformation in response to ATD and circulating antibodies against neutrophils were significant in patients with ATD-induced agranulocytosis compared with control patients (6). Using direct immunofluorescence tests, a transient autoantibody response in patients with agranulocytosis has been shown to be induced by propylthiouracil (7). This ATD-induced specific immune-mediated response reacted not only with mature granulocytes, but also affected mature blood cells and myeloid progenitor cell growth. These results suggest an immune-mediated mechanism rather than direct toxic effects of the drug. The immune-mediated destruction of mature neutrophils was the first mechanism to be identified as a cause of ATD-induced agranulocytosis. Sprikkelman *et al* (8) described four different immunological mechanisms that may be responsible (8). Firstly, antibodies may develop against the antithyroid drug when it is bound to the cell membrane of the granulocyte, resulting in an accelerated destruction of the granulocyte. Secondly, antibodies may target the drug/metabolite complex that has been adsorbed to the neutrophil granulocyte in the presence of plasma component. Thirdly, the drug may trigger the production of auto-antibodies. Finally, the interaction of a granulocyte antigen and drug may induce the production of antibodies. In addition, other immunological reactions include the immunoglobulin E (IgE)-mediated hypersensitivity reaction, drug-induced IgG and IgM responses and antineutrophil cytoplasm antibody (ANCA)-associated immune injury, which may contribute to agranulocytosis (9–11). In the present case, the patient was diagnosed with agranulocytosis after MMI was discontinued for four months. A review of previous studies found no similar report. Although ATD-induced agranulocytosis is considered to be mediated primarily by immunological mechanisms, this does not explain the pathogenesis of the present patient, and further investigation is required.

In conclusion, ATD-induced agranulocytosis is a rare but potentially fatal idiosyncratic reaction. It is not usually possible to predict which patients are likely to be susceptible, so conducting a routine complete blood cell count is suggested, and informing the patient of the common symptoms of agranulocytosis may contribute to an early diagnosis. Admission to hospital and treatment with GCSF may accelerate neutrophil recovery. The present case report aims to increase the awareness of the onset agranulocytosis from discontinued MMI treatment, and warn that MMI should be used with caution.

## Figures and Tables

**Table I:** Changes of selective indices observed in the patient during treatment.

Parameters studied	Day-1	Day 0	Day 1	Day 2	Day 3	Day 4	Day 5	Day 6	Day 10
WBC count (x10^9^/l)	1.28	0.50	0.50	0.57	0.92	1.42	2.22	5.86	6.53
Neutrophil ratio (%)	3.5	2.2	1.3	3.9	4.7	5.2	30.1	42	65
Neutrophil count (x10^9^/l)	0.05	0.0	0.01	0.02	0.04	0.07	0.67	2.46	4.53
HGB (g/l)	134	134	111	111	111	107	121	117	119
PLT (x10^9^/l)	231	190	153	146	147	125	133	110	130
Free T_3_ (pmol/l)			12.58						
Free T_4_ (pmol/l)			80.45						
TSH (mIU/l)			0.01						

WBC, white blood cell; HGB, hemoglobin; PLT, platelet; TSH, thyroid-stimulating hormone.

## References

[1] Andres E, Dali-Youcef N, Serraj K, Zimmer J (2009). Recognition and management of drug-induced blood-cytopenias: the example of idiosyncratic drug-induced thrombocytopenia. Expert Opin Drug Saf.

[2] Tajiri J, Noguchi S (2004). Antithyroid drug-induced agranulocytosis: special reference to normal white blood cell count agranulocytosis. Thyroid.

[3] Tamai H, Takaichi Y, Morita T, Komaki G (1989). Methimazole-induced agranulocytosis in Japanese patients with Graves’ disease. Clin Endocrinol (Oxf).

[4] Waldhauser L, Uetrecht J (1991). Oxidation of propylthiouracil to reactive metabolites by activated neutrophils. Implications for agranulocytosis. Drug Metab Dispos.

[5] Pisciotta AV (1973). Immune and toxic mechanisms in drug-induced agranulocytosis. Semin Hematol.

[6] Wall JR, Fang SL, Kuroki T, Ingbar SH, Braverman LE (1984). In vitro immunoreactivity to propylthiouracil, methimazole, and carbimazole in patients with Graves’ disease: a possible cause of antithyroid drug-induced agranulocytosis. J Clin Endocrinol Metab.

[7] Toth EL, Mant MJ, Shivji S, Ginsberg J (1988). Propylthiouracil induced agranulocytosis: an unusual presentation and a possible mechanism. Am J Med.

[8] Sprikkelman A, de Wolf JT, Vellenga E (1994). The application of hematopoietic growth factors in drug-induced agranulocytosis: a review of 70 Cases. Leukemia.

[9] Sun MT, Tsai CH, Shih KC (2009). Antithyroid drug-induced agranulocytosis. J Chin Med Assoc.

[10] Akamizu T, Ozaki S, Hiratani H (2002). Drug-induced neutropenia associated with anti-neutrophil cytoplasmic antibodies (ANCA): possible involvement of complement in granulocyte cytotoxicity. Clin Exp Immunol.

[11] Harper L, Chin L, Daykin J (2004). Propylthiouracil and carbimazole associated- antineutrophil cytoplasmic antibodies (ANCA) in patients with Graves’ disease. Clin Endocrinol (Oxf).

